# microRNAs: The Short Link between Cancer and RT-Induced DNA Damage Response

**DOI:** 10.3389/fonc.2014.00133

**Published:** 2014-06-04

**Authors:** Christopher M. Wright, Tu Dan, Adam P. Dicker, Nicole L. Simone

**Affiliations:** ^1^Department of Radiation Oncology, Kimmel Cancer Center, Jefferson Medical College, Thomas Jefferson University, Philadelphia, PA, USA

**Keywords:** radiation, DNA damage/response, microRNA, cancer, therapeutic target, oxidative stress, double-strand breaks, carcinogenesis

DNA damage response (DDR) networks have long been noted to be implicated in cell death induced via ionizing radiation (1). These DNA damage sensing and signaling pathways establish control through cell cycle checkpoints, cellular senescence, and apoptosis (2). When functioning properly, DDR networks act as a barrier against tumor growth while maintaining genome integrity. New discoveries have unveiled specific roles of proteins in DDR networks, which may serve as potential therapeutic targets and sensitizers to ionizing radiation (3).

Unfortunately, although a clear connection has been established between dysfunctional DDR networks and malignancy, clinical trials targeting these pathways in the oncology realm have shown limited efficacy to date (4, 5). Lapsed regulation of DDR pathways in malignancy allows cells to bypass cellular checkpoints and progress through the cell cycle with stalled replication forks, incomplete DNA replication, and other forms of DNA damage (6). This genomic instability is propagated through cellular generations resulting in a neoplastic phenotype. A number of specific pathognomonic DDR defects have been identified in a number of cancers, including the mismatch repair protein MSH2 in colorectal cancer and the homologous recombination proteins BRCA1 and BRCA2 in breast and ovarian cancers (7, 8). Recent evidence suggests DDR mishaps may occur at an early stage in some precancerous lesions, double-strand break (DSB) markers such as nuclear gamma-H2AX are significantly elevated (9).

To further understand the role of DDR in malignancy, attention can be turned to the investigation of microRNAs (miRs), as another component of the DDR machinery in post-transcriptional gene regulation (10). miRs are small, non-coding RNA molecules that are complementary to one or more messenger RNA molecules (mRNA) (11). This specific pairing leads to the translational inhibition and degradation of the target mRNA. Global dysregulation of miRNAs is frequently observed in malignancy and patterns of dysregulation seem to be dependent on cancer type (12). More recently, it has been demonstrated that miR expression is regulated by DNA lesions and DDR proteins (13). It is suggested that miRs may play a regulatory role in an intermediary timeframe, in between rapid post-translational protein modifications and delayed transcriptional activation of target genes (14).

Our laboratory has previously shown that normal human fibroblasts exhibit unique miRNA signatures when exposed to exogenous agents that induce oxidative or genotoxic stress (15). A time course after exposure showed changes in 17 miR species following exposure to radiation, 23 after H_2_O_2_ treatment, and 45 after etoposide treatment. The miR signatures varied with direct (etoposide) and indirect (H_2_O_2_) effects (Figure 1). Eight miRs were altered specifically by radiation and etoposide, suggesting these might be used to discern direct DNA damage due to radiation. Alternatively, two miRs were altered with radiation and H_2_O_2_, suggesting these could comprise a signature of indirect DNA damage. These arrays did not demonstrate any significantly altered miRs that were unique to radiation alone. Interestingly, production of reactive oxygen species (ROS) increased with increasing doses of radiation. Additionally, pre-treatment with the thiol antioxidant cysteine decreased both ROS production and reversed the changes in the miRNA signature in response to irradiation.

**Figure 1:**
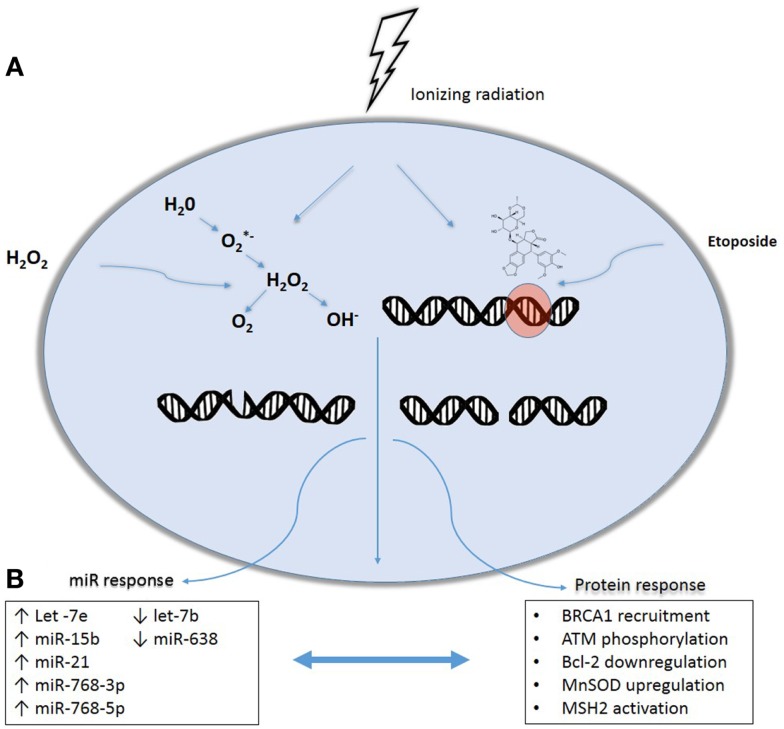
**Effects of cytotoxic stressors on microRNAs and protein expression interplay**. DNA damage may occur following exposure to genotoxic agents including ionizing radiation, etoposide, and hydrogen peroxide (H_2_O_2_) exposure. Ionizing radiation causes DNA damage both directly, by energetic disruption of DNA integrity, and indirectly, as a result of the formation of intracellular free radicals resulting in both double and single strand breaks. Similarly, DNA damage by H_2_O_2_ is produced by reactive oxygen species formation while etoposide generates double-strand breaks mimicking the direct DNA damage caused by radiation. **(A)** In response, damage-sensing repair molecules are recruited to the site of DNA damage, triggering multiple protein-mediated repair cascades. In parallel, DNA damage activates the processing of miRNA precursors, eventually leading to expression of a common miRNA signature produced in response to genotoxic stress. Post-transcriptional regulation of mRNAs mediated by these induced miRNAs plays a fundamental role in adjusting DDR machinery **(B)**, with complex interplay between protein and miRNA expression.

The miRs affected in our study are reflective of more recent literature investigating individual miRs that are altered in response to DDR (16). In fact, they are implicated in more mechanistic studies dealing with homologous recombination, non-homologous end joining, and base excision repair (17, 18). Post-transcriptional regulation of mRNAs mediated by miRs plays a fundamental role in adjusting DDR machinery. miR-421 in neuroblastoma and HeLa cells downregulates ATM kinase, which is a crucial integrator of DNA DSBs repair machinery (19). Ectopic expression of miR-421 leads to S-phase cell cycle checkpoint changes and an increase in radiosensitivity. Although it has not been clearly demonstrated that miRs directly mediate the choice between homologous recombination and NHEJ-mediated repair of a DSB, evidence suggests that miRs are at least intimately involved by targeting factors that belong to a specific pathway. Expression of miR-182 directly downregulates BRCA1 and defers from homologous recombination (20). Alternatively, the expression of miR-101 and miR-34a would downregulate DNA-PKcs and p53 binding protein 1, respectively, impeding the NHEJ repair pathway (21, 22). Other miRNAs, such as miR-34, miR-521, miR-21, have been shown to regulate the expression of important DDR network proteins BCL2, manganese superoxide dismutase (MnSOD), and MSH2, respectively (23–25).

Due to the miRNA regulation of DDR machinery and to the clear connection between DDR dysregulation and a neoplastic phenotype, we believe miRs could define the relationship between cancer and DDR. Our laboratory’s studies suggest that miRs serve as integrators of the cellular response to ROS and DNA strand breaks, both of which are results of ionizing radiation. It is our opinion that further investigation of miR impact on cellular sensitivity to DNA-damaging agents could elucidate therapeutic targets to combat cancer, as miRs may provide the link between DDR and malignancy.

## Conflict of Interest Statement

The authors declare that the research was conducted in the absence of any commercial or financial relationships that could be construed as a potential conflict of interest.
